# Color-Coded Front-of-Pack Nutrition Labels—An Option for US Packaged Foods?

**DOI:** 10.3390/nu9050480

**Published:** 2017-05-10

**Authors:** Elizabeth K. Dunford, Jennifer M. Poti, Dagan Xavier, Jacqui L. Webster, Lindsey Smith Taillie

**Affiliations:** 1 Food Policy Division, The George Institute for Global Health, University of New South Wales, Sydney, NSW 2042, Australia; jwebster@georgeinstitute.org.au; 2 Carolina Population Center, The University of North Carolina at Chapel Hill, Chapel Hill, NC 27516, USA; poti@unc.edu (J.M.P.); smithlp@email.unc.edu (L.S.T.); 3 Department of Nutrition, The University of North Carolina at Chapel Hill, Chapel Hill, NC 27516, USA; 4 Label Insight, Chicago, IL 60661, USA; dxavier@labelinsight.com; 5 Office of the Chief Scientist, The George Institute for Global Health, University of New South Wales, Sydney, NSW 2042, Australia

**Keywords:** food labels, processed foods, public health nutrition, nutrient profiling

## Abstract

The implementation of a standardized front-of-pack-labelling (FoPL) scheme would likely be a useful tool for many consumers trying to improve the healthfulness of their diets. Our objective was to examine what the traffic light labelling scheme would look like if implemented in the US. Data were extracted from Label Insight’s Open Access branded food database in 2017. Nutrient levels and the proportion of products classified as “Red” (High), “Amber” (Medium) or “Green” (Low) in total fat, saturated fat, total sugar and sodium for food and beverage items were examined. The proportion of products in each category that had each possible combination of traffic light colors, and met the aggregate score for “healthy” was examined. Out of 175,198 products, >50% of all US packaged foods received a “Red” rating for total sugar and sodium. “Confectionery” had the highest mean total sugar (51.9 g/100 g) and “Meat and meat alternatives” the highest mean sodium (781 mg/100 g). The most common traffic light label combination was “Red” for total fat, saturated fat and sodium and “Green” for sugar. Only 30.1% of products were considered “healthy”. A wide variety (*n* = 80) of traffic light color combinations were observed. A color coded traffic light scheme appears to be an option for implementation across the US packaged food supply to support consumers in making healthier food choices.

## 1. Introduction

Americans today have access to more nutrition information than any previous generation. The US food supply is dominated by packaged food and beverage products, with a huge array of information available on product packaging about the healthfulness of each item. In the US, the Food and Drug Administration (FDA) mandates the declaration of eight nutrients on the back-of pack to educate consumers on what is in the foods they buy [1]. Additionally, manufacturers can display health and nutrient content claims on the front-of-pack (FOP), and can use a wide variety of logos, graphics, and wording to encourage consumers to buy their products. Despite this huge amount of information available to help consumers make healthier food choices, intake of energy dense, nutrient poor foods, and subsequent levels of obesity and non-communicable diseases (NCDs) in the US population [2] have continued to increase dramatically over the past few decades [3].

FOP labelling has been identified as one potential policy lever to increase the healthfulness of foods and beverages Americans buy and eat. In part this is because this type of labelling can encourage manufacturers to reformulate their products to be healthier, and in part because they can make the identification of healthier food choices more obvious to consumers and help alleviate the consumer-driven mandate for greater transparency. In 2010, the US Institute of Medicine launched the first phase of its report on FOP nutrition labels and provided some early recommendations about what might be appropriate in the US [4]. The report recommended that any scheme developed for use in the US focus on calories, saturated fat, trans fat, and sodium (with sugar not considered in this report). However, when the phase 2 report was released in 2011 [5], the recommendations included added sugars and recommended a point system for evaluating the amounts of nutrients in processed foods. However, after supposed pressure from the food industry [6], the FDA did not proceed with development of a standardized FOP nutrition label scheme for the USA, meaning that consumers still do not have a consistent tool to support them to make healthier food choices.

A large number of FOP labelling schemes have been developed and implemented in other parts of the world [7], all aiming to help consumers make healthier food and beverage choices in the supermarket. Despite governments, food manufacturers, researchers and retailers having explored which one of many various FOP labelling schemes consumers may prefer [8,9,10,11], there remains no consensus among stakeholders on the way forward. More importantly, there has been little research undertaken exploring what different FOP labelling schemes would look like when applied to US foods. The UK Department of Health’s voluntary color-coded FOP labelling scheme (first launched in 2009), which uses traffic light colors to indicate whether a food is “Red” (High) “Amber”, (Medium) or “Green” (Low) in total fat, saturated fat, total sugars and sodium (salt) [12], has consistently come out as one of the top FOP labelling schemes in both consumer preference and its ability to support consumers in identifying healthier choices [13]. One study compared consumer understanding of the US food industry-developed “Facts Up Front” FOP labelling scheme to the traffıc light system and found that traffıc light labels best assisted individuals in judging the nutritional profiles of foods and beverages [10]. A separate review from 2014 found that the traffic light system was the most understood by US consumers [14].

However, to date there have been no studies examining what the implementation of this type of FOP labelling scheme would mean for the US food supply. Firstly, it is not known what proportion of US products would receive “Red”, “Amber” or “Green” lights for each nutrient, so as to understand the potential for consumer behaviour change and product reformulation. Secondly, the distribution of the nutritional profile of products receiving these traffic light labels is also unknown. As such, the aim of the current study was to examine what the traffic light labelling scheme would look like as a FOP labelling option for the USA with regards to the proportion and nutritional profile of products covered.

## 2. Materials and Methods

Data for this study are from the largest publically available branded food composition database in the US. Label Insight, who provide data solutions that allow transparency between consumer packaged goods brands, retailers and consumers, launched the Open Data initiative in 2017. The initiative provides researchers with open access to granular food composition data not previously available to the research community. Researchers are granted the freedom to publish their findings based on Label Insight’s data without restriction. The database is updated daily, and contains information on more than 290,000 barcoded food and beverage items (representing >85% of all products sold in the US food supply over the past three years).

### 2.1. Data Collection

We used Label Insight data extracted in January 2017. Nutrient data were extracted for 295,606 barcoded food and beverage items from the Label Insight portal. The following fields of information were extracted: Universal Product Code (UPC), brand name, product description, serving size, energy content (calories/serve), total fat (g/serve), saturated fat (g/serve), total sugar (g/serve) and sodium (mg/serve). After removal of products with duplicate UPCs (*n* = 285), bulk items not designed for individual consumer purchase (*n* = 835), products with implausible nutrient values (defined as >100 g of total fat, saturated fat or sugars per 100 g) (*n* = 298), products that did not display a nutrition facts panel (NFP) (such as tea, coffee, fresh produce etc.) (*n* = 82,248), products that were considered outliers following range checks at the food category level (*n* = 5977) and products that were considered to be variety packs with multiple NFPs or single ingredient items that cannot be reformulated (such as sugar) (*n* = 30,899), there were 175,198 products remaining for analysis.

### 2.2. Food Categorization

Foods were categorized into one of 13 major food categories and 45 food subcategories based on the Global Food Monitoring Group’s categorization system [15], a global system used to examine the healthfulness of national food supplies. Supplementary Table S1 shows a description of each category.

### 2.3. Assignment of Traffic Light Criteria

Foods were classified as “Red” (High), “Amber” (Medium) or “Green” (Low) in total fat, saturated fat, total sugar, and sodium, based on the UK Department of Health traffic light criteria. The criteria can be seen in Table 1 and Table 2. The criteria are based on both per 100 g/mL and per serving nutrient values and two separate sets of criteria exist for foods and beverages. As nutrition labels in the US display only per serving nutrient values, these were converted to per 100 g by multiplying the per-serving nutrient value by 100 and dividing by the serving size. Each food category was identified as being either “food” or “beverage” in order to apply the appropriate criteria.

### 2.4. Overall Healthfulness

To examine overall healthfulness, a previously developed traffic light label aggregate score was also assigned to each product: one point was assigned for every green light, two for every amber light, and three for every red light, giving a total possible score of between four and 12. Foods were considered “healthy” if the total score based on the level of four key nutrients was less than seven [16]. Scores were examined overall, separately for foods and beverages, and by each major food category and subcategory.

### 2.5. Statistical Analysis

All analyses were undertaken using Stata version 14.1 (StataCorp, College Station, TX, USA). Descriptive statistics were used to describe the proportion of products classified as “Red” (High), “Amber” (Medium) or “Green” (Low) in total fat, saturated fat, sugar and sodium using the UK Department of Health criteria. Results were stratified by categories and subcategories. The proportion of products in each major category that had each possible combination of traffic light colors was also examined (80 possible combinations) to determine if consumers would have sufficient choice if traffic light labels were to be implemented in the US. The mean, median and range of values for total fat, saturated fat, sugar and sodium per 100 g/mL were also calculated by each major category and subcategory. Within products that received a “Red” (High) traffic light for total fat, saturated fat, total sugars and sodium, the proportion that received a “Red” (High) rating due to exceeding the serving size cut-offs versus the per 100 g/mL criteria was evaluated by both food and beverages separately, and by major category.

## 3. Results

### 3.1. Overall Results

More than 40% of all US packaged food products received a “Red” (High) rating for sodium, total fat, saturated fat and total sugars (Table 3). More than 50% received a “Red” (High) rating for both total fat and sodium. Total sugar was the only nutrient for which a larger proportion of products received a “Green” (Low) rating than a “Red” (High) rating. A much smaller proportion of products received an “Amber” (Medium) rating for all nutrients. When examining the healthfulness of food and beverage options using the traffic light aggregate score method, only 30.1% of all products were considered “healthy”, ranging from only 0.3% of “Edible oils” to 81.1% of “Beverages” (Supplementary Table S2). However, results differed greatly when results were split into food and beverage items. “Beverages” had a larger proportion of products receiving a “Red” (High) rating for total sugars compared to “Food” (59.2% versus 39%) and a much lower proportion of products receiving a “Red” (High) for sodium (8.9% versus 54.0%). Similarly, “Beverages” had a much larger proportion of products classified as “healthy” using the traffic light aggregate scores (66.1% versus 27.5%). Results also varied when products were broken down into each major food category and subcategory (Supplementary Table S3).

### 3.2. Total Fat and Saturated Fat

**Figure 1:**
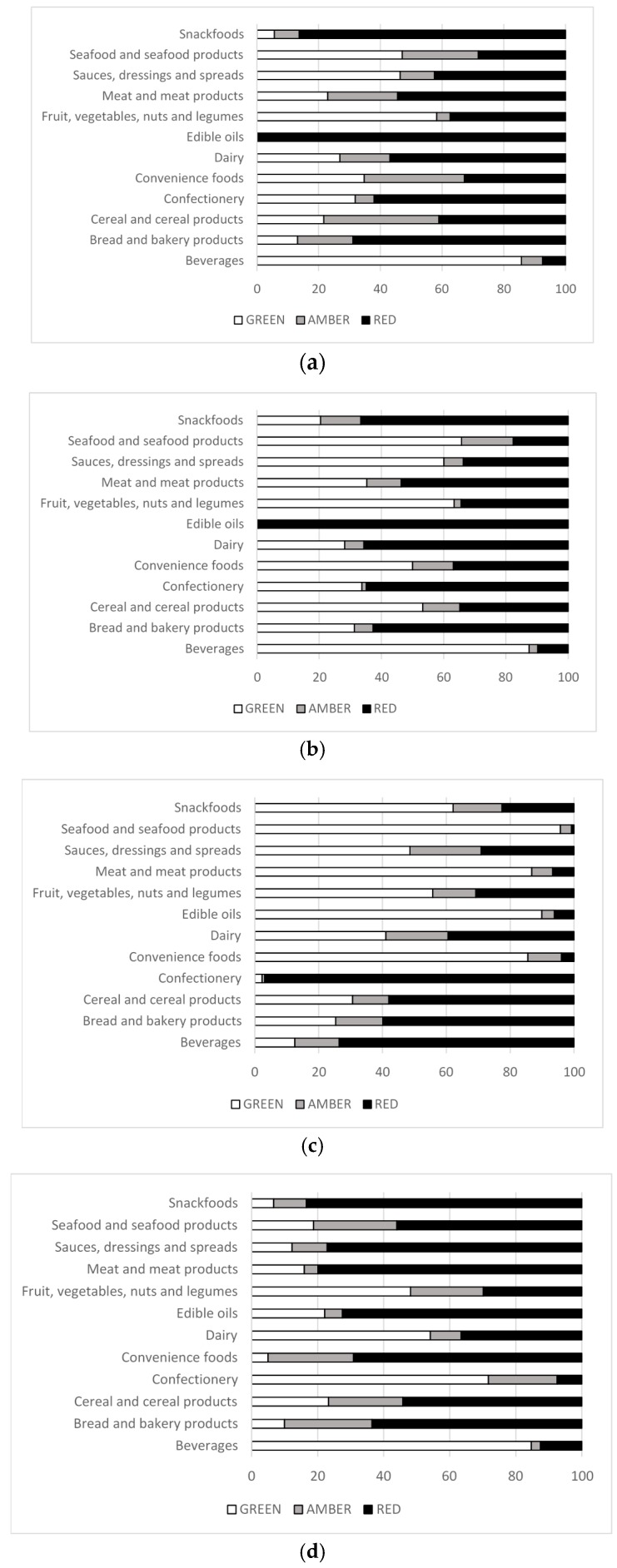
Proportion of products in each major food category meeting traffic light criteria: (**a**) Total fat; (**b**) Saturated fat; (**c**) Total sugars; (**d**) Sodium.

The total fat and saturated fat content for all products both overall and by food category is shown in Table 4. “Edible oils” had the highest mean total fat content (68.7 g/100 g) followed by “Snack foods” (22.9 g/100 g). “Edible oils” also had the highest mean saturated fat content (31.0 g/100 g) and “Confectionery” the second highest (10.5 g/100 g). The “Beverages” category had the lowest mean total fat content (1.2 g/100 g), followed by “Seafood” (1.3 g/100 g). The “Beverages” category had the highest proportion of products receiving a “Green” (Low) rating for total fat and saturated fat (86% and 87% respectively), and the “Edible oils” category the highest proportion of products receiving a “Red” (High) rating for total fat and saturated fat (100% and 99% respectively) (Figure 1a,b). Within product subcategories some variation in results was seen. For example, within the “Bread and bakery” major category, only 5% of products in the Biscuits and crackers and Cakes, muffins and pastry subcategories received a “Green” (Low) rating for total fat compared to 38% of products in the Bread subcategory (Supplementary Table S3). 34% of food products and 6% of beverage products received a “Red” (High) traffic light for total fat as they exceeded the serving size cut-off of >21 g/serving and >10.5 g/serving respectively (Supplementary Table S4). For saturated fat, 29% of foods and 28% of beverages received a “Red” (High) traffic light due to exceeding the serving size cut-offs (>6 g and >3 g/serving respectively). When examining specific categories, “Convenience foods” had the highest proportion of products receiving a “Red” (High) traffic light for total fat due to exceeding the serving size cut-off (Supplementary Table S5).

### 3.3. Total Sugars

“Confectionery” was the category with the highest mean total sugar content (51.9 g/100 g) (Table 4) and had the largest proportion of products receiving a “Red” (High) rating (97%) for total sugar (Figure 1c). The “Beverages” category also had a high proportion of products receiving a “Red” (High) rating for sugar (74%). However, within “Beverage” subcategories the proportion of products receiving a “Red” (High) traffic light for sugar varied. For example, less than 50% of Waters and Electrolyte drinks had a “Red” (High) traffic light for total sugar, whereas for Soft drinks and Juices it was 87% and 80% respectively (Supplementary Table S3). Interestingly, although the “Cereal and cereal products” category overall had 58% of products receiving a “Red” (High) rating for total sugar, 95% of Cereal and nut-based bars and 76% of Breakfast cereal products received a “Red” (High) traffic light for sugar. The “Seafood” category had the lowest mean sugar content (0.8 g/100 g), followed by “Edible oils” (2.0 g/100 g) and “Meat and meat products” (2.7 g/100 g). “Seafood” also had the highest proportion of products receiving a “Green” (Low) rating for total sugar. Just under two thirds (29%) of food products and 49% of beverage products received a “Red” (High) traffic light for total sugars as they exceeded the serving size cut-off of >27 g/serving and >13.5 g/serving respectively (Supplementary Table S4). “Convenience foods” and “Seafood” products had the highest proportion of products receiving a “Red” (High) traffic light for total sugars due to exceeding the serving size cut-off, and 50% of beverages (Supplementary Table S5). “Confectionery” had 98% of products receiving a “Red” (High) traffic light for total sugars solely by using the per 100 g criteria (Supplementary Table S5).

### 3.4. Sodium

“Meat and meat products” had the highest mean sodium content (781 mg/100 g) (Table 4), with “Sauces, dressings and spreads” a close second (751 mg/100 g). The “Snack foods” category had the highest proportion of products receiving a “Red” (High) rating for sodium (83%), and “Confectionery” the lowest (7%) (Figure 1d). Only three categories had >50% of products receiving a “Green” (Low) rating for sodium; “Confectionery”, “Beverages” and “Dairy”. However, the “Dairy” category was the category with the most variation in sodium between subcategories, with 90% of Cheese products having a “Red” (High) rating for sodium and all other “Dairy” subcategories having <10% (Supplementary Table S3). More than half (54%) of food products and 21% of beverage products received a “Red” (High) rating for sodium as they exceeded the serving size cut-off of >720 mg/serving and >360 mg/serving respectively (Supplementary Table S4). “Confectionery” had the highest proportion of products receiving a “Red” (High) traffic light for sodium due to exceeding the serving size cut-off, and “Edible oils” had the largest proportion receiving a “Red” (High) traffic light for sodium solely using the per 100 g criteria (Supplementary Table S5).

### 3.5. Traffic Light Label Combinations

Every possible traffic light color combination was observed across foods and beverages. The most common traffic light color combination for all US packaged foods was “Red” (High) for total fat, saturated fat and sodium and “Green” (Low) for sugar. (Supplementary Table S6). This was the most common combination for five major food categories; “Convenience foods”, “Dairy”, “Edible oils”, Meat and meat products” and “Snack foods”. “Bread and bakery products” was the only category to have the combination of “Red” (High) traffic lights as the most common for all nutrients. The common color combination for each major category had at least one “Red” (High) nutrient. Beverages received a “Red” (High) traffic light for total sugars, with “Green” (Low) for all other nutrients. Only 5% of products received a “Green” (Low) rating for every nutrient, with an additional 8% receiving three “Green” (Low) ratings and one “Amber” (Medium) rating (Supplementary Table S6).

## 4. Discussion

This is the first study to demonstrate what the color coded “traffic light” FOP labelling system would look like if implemented in the US food supply. We found that more than 40% of all US packaged food and beverage products received a “Red” (High) rating for total fat, saturated fat, total sugar and sodium, and that more than 50% of packaged food and beverage items in our analysis were considered “unhealthy”. However, the traffic light labelling criteria also demonstrate the substantial variability in levels of total fat, saturated fat, total sugar and sodium both within and across product categories as well as variability in the overall “healthfulness” of products and a variety of traffic light color combinations indicating it could be a useful tool to support US consumers to make healthier packaged food and beverage choices.

Assuming that consumers would interpret “Red” traffic light labels to mean “less healthy” and “Green” to mean “healthy”, results from this analysis show that there would be a range of healthy products for consumers to choose from across each food and beverage category as packaged products would exist in the US market with every possible traffic light color combination. This lends support for the implementation of a traffic light label format to help guide consumers to choose healthier food choices and is supported by research from other developed countries which has shown that the application of color-coded nutrient criteria provides a range of traffic light color combinations for consumers to choose from across various food categories [17].

It is not only through consumer behavior change that the implementation of a traffic light labelling format could have an effect on the healthfulness of consumer packaged food purchases, but there is evidence that manufacturers make efforts to reformulate their products to meet criteria for different FOP labelling schemes [4,18]. Further support for this is seen in a UK study which found that consumers were willing to pay more for a change from a “Red” to a “Green” label, compared with from “Amber” to “Green”, suggesting an aversion to “Red” labels [19]. As we observed that such a large proportion of products with a “Red” (High) traffic light rating only received the “Red” (High) rating due to exceeding the per serving guidelines (for example, 54% of food products receiving a “Red” traffic light for sodium actually had a per 100 g/mL value within the “Amber” or “Green” criteria, however the per serving criteria were exceeded), there appears to be scope for US manufacturers to reformulate products not only in terms of per 100 g/mL composition, but also in relation to the serving sizes they offer consumers. The increasing portion sizes available to US consumers is well-known [20,21], and the implementation of a FOP labelling scheme that uses both per 100 g/mL and per serving criteria may provide a stronger impetus for manufacturers to reformulate their products and reduce portion sizes.

The debate over which FOP labelling scheme is the most appropriate for the US is likely to continue for the foreseeable future. A number of other countries around the world are exploring the potential for color-coded schemes like the traffic light labelling scheme, adding further support for its implementation in the US. For example, color-coding has been proposed in South Africa, where a draft regulation sets requirements for a voluntary traffic light label for total sugar, fat, saturated fat and total sodium [6]. Korea was the first Asian country to release recommendations for voluntary traffic light labels on children’s food, and color coding is also under consideration in India, where the government is looking at traffic light labels as part of a group of measures to address obesity-related NCDs [6]. France has also recently announced the implementation of a five-category color label [22].

The fact that more than 50% of all products were “Red” (High) in sodium is an interesting finding. Sodium reduction has been a focus of national health institutions through both the National Salt Reduction Initiative [23] and the recently proposed FDA salt reduction targets [24]. Recent studies have shown that sodium levels in the US food supply have been decreasing [23], however it appears that the majority of products are still providing excessive levels of sodium to the American diet. With a growing amount of literature showing the huge potential for sodium reduction in processed foods to help reduce the burden of cardiovascular disease in the population [25], and our results showing more than half of all products are high in sodium, a FOP labelling scheme such as traffic light labelling could potentially push manufacturers to reduce the levels of sodium in their products towards “better” traffic light colors, and hence help reduce population dietary intake of sodium.

Research in this area to date has focused on consumer understanding and use of various FOP labelling schemes, however there is limited research to examine whether these schemes also support the purchase of healthier food choices by consumers. Most recently, studies have appeared which utilise smartphone technology to understand whether consumer use of FOP labelling schemes translates to healthier food choices. For example, the Starlight trial undertaken in New Zealand compared the mean healthfulness of all packaged food purchases over a four week intervention period and found that traffic light label users had significantly healthier food purchases than users of the standard back-of-pack information [26]. A similar study is currently underway in Australia comparing the healthfulness of food purchases using five different forms of labelling via a consumer’s smartphone [27].

The analysis for this research used nutritional values reported on product labels and so may not accurately represent what is in the foods. However prior studies suggest that nutrition label data are generally accurate and within the FDA limits [28]. A limitation of the traffic light label scheme is its focus on nutrients, rather than food-based or diet pattern-based dietary guidelines [29]. However, there remains no consensus in the literature as to whether nutrient-based or food-based nutrient profiling criteria for FOP labelling are both meaningful for consumers, and easy for industry to implement. Our analysis included only packaged food products, with fresh meat and produce excluded, as well as single ingredient products such as sugar and eggs. As such, it is difficult to quantify the healthfulness of the overall US food supply.

## 5. Conclusions

With more than two thirds of the diet of the average American deriving from packaged food and beverages [1], the implementation of a standardized FOP labelling scheme would likely be a useful tool for many consumers trying to improve the healthfulness of their diets. We observed a wide variation in traffic light label color combinations for US packaged foods. A scheme similar to the color-coded traffic light scheme appears to be one option to consider for implementation across the US packaged food supply to support consumers in making healthier food choices.

## Figures and Tables

**Table 1:** Colour coded criteria for food items [12].

	Green (Low)	Amber (Medium)	Red (High)	
Total fat	≤3.0 g/100 g	>3.0 to ≤17.5 g/100 g	>17.5 g/100 g	>21 g/serving
Saturated fat	≤1.5 g/100 g	>1.5 to ≤5.0 g/100 g	>5.0 g/100 g	>6.0 g/serving
Total sugars	≤5.0 g/100 g	>5.0 to ≤22.5 g/100 g	>22.5 g/100 g	>27 g/serving
Sodium	≤120 mg/100 g	>120 to ≤600 mg/100 g	>600 mg/100 g	>720 mg/serving

**Table 2:** Colour coded criteria for beverage items [12].

	Green (Low)	Amber (Medium)	Red (High)	
Total fat	≤1.5 g/100 mL	>1.5 to ≤8.75 g/100 mL	>8.75 g/100 mL	>10.5 g/serving
Saturated fat	≤0.75 g/100 mL	>0.75 to ≤2.5 g/100 mL	>2.5 g/100 mL	>3 g/serving
Total sugars	≤2.5 g/100 mL	>2.5 to ≤11.25 g/100 mL	>11.25 g/100 mL	>13.5 g/serving
Sodium	≤120 mg/100 mL	>120 to ≤300 mg/100 mL	>300 mg/100 mL	>400 mg/serving

**Table 3:** Proportion of US food and beverage products with each color coded nutrient.

	Total Fat		Saturated Fat		Sugars		Sodium	
	*n*	%	*n*	%	*n*	%	*n*	%
Food								
Green	50,753	31.1	68,910	42.2	79,288	48.6	45,568	27.9
Amber	24,580	15.1	11,369	7.0	20,224	12.4	29,561	18.1
Red	87,889	53.9	82,943	50.8	63,710	39.0	88,093	54.0
Beverages								
Green	7992	67.2	8743	73.6	1692	14.2	10,360	87.2
Amber	2354	19.8	812	6.8	3163	26.6	468	3.9
Red	1540	13.0	2331	19.6	7031	59.2	1058	8.9
All foods								
Green	58,781	33.6	77,689	44.4	81,028	46.3	55,952	31.9
Amber	26,941	15.4	12,187	7.0	23,391	13.4	30,077	17.2
Red	89,455	51.1	85,301	48.7	70,758	40.4	89,148	50.9

**Table 4:** Mean and range of nutrient levels in each major food category.

		Total Fat (g/100 g)		Saturated Fat (g/100 g)		Total Sugars (g/100 g)		Sodium (mg/100 g)	
Food category	*n*	Mean	Range	Mean	Range	Mean	Range	Mean	Range
Beverages	7075	1.7	0–56.3	1.2	0–37.5	16.3	0–100.0	104	0–3000
Bread and bakery products	26,804	14.2	0–75.0	5.4	0–52.2	20.6	0–97.0	396	0–3069
Cereal and cereal products	8855	9.6	0–53.6	2.5	0–40.7	17.4	0–75.0	427	0–6000
Confectionery	15,394	18.3	0–100.0	10.5	0–64.3	51.9	0–100.0	107	0–2422
Convenience foods	14,375	6.7	0–64.3	2.3	0–24.2	3.1	0–63.3	541	0–29,000
Dairy	25,815	13.7	0–85.7	8.2	0–50.0	10.2	0–100.0	335	0–3929
Edible oils and emulsions	357	68.7	0–100.0	31.0	0–71.4	2.0	0–47.5	517	0–1950
Seafood	4876	6.0	0–84.9	1.3	0–25.9	0.8	0–38.3	464	0–10,625
Foods for specific dietary use	2053	13.1	0–46.4	4.9	0–27.7	18.1	0–100.0	267	0–1282
Fruit, vegetables, nuts and legumes	26,861	15.5	0–100.0	2.5	0–66.7	13.6	0–95.2	252	0–6300
Meat and meat products	12,374	13.6	0–100.0	4.7	0–38.5	2.7	0–57.1	781	0–8375
Sauces, dressings and spreads	18,805	14.4	0–100.0	2.6	0–64.3	10.2	0–100.0	751	0–19,286
Snack foods	11,464	22.9	0–91.7	5.4	0–60.0	10.3	0–98.9	651	0–7800
TOTAL	175,108	13.8	0–100.0	4.8	0–71.4	15.3	0–100.0	424	0–29,000

## Supplementary Materials

The following are available online at www.mdpi.com/2072-6643/9/5/480/s1, Table S1: Global Food Monitoring Group food categorization system, Table S2: Traffic light label aggregate scores for US products, Table S3: Proportion of US food and beverage products meeting each color coded criteria by food subcategory, Table S4: Nutrient levels per 100 g for US products that receive a “Red” traffic light rating, Table S5: Nutrient levels per 100 g for US products that receive a “Red” traffic light rating by food subcategory, Table S6: Proportion of traffic light color combinations for each major food category.
