# Supplementary material for: Color-Coded Front-of-Pack Nutrition Labels—An Option for US Packaged Foods?
Source: Nutrients. 2017 May 10;9(5):480. doi: 10.3390/nu9050480 (PMC5452210; doi:10.3390/nu9050480)
Supplement: Supplementary file 1 [file nutrients-09-00480-s001.pdf]

Supplementary tables

Supplementary Table 1: Global Food Monitoring Group food categorization system

Food group	Food category	Description
**Beverages**	Juices	Fresh and ambient fruit and vegetable juices
	Soft drinks	Sugar-sweetened and artificially-sweetened soft drinks
	Electrolyte drinks	Sports electrolyte drinks
	Waters	Plain and flavored waters
	Coffee and tea	All coffee and tea products
	Powdered beverages	All beverage mixes
**Bread and bakery products**	Bread	White, wholemeal and mixed grain/seed sliced bread and rolls
		Fruit bread and fruit-based muffins/rolls
		Wraps and other flatbread products
		Turkish pide, bagels, English-style muffins, crumpets, pizza bases and other plain bread-based products
	Biscuits and cookies	Filled and unfilled sweet biscuits
		Flavored and plain crisp bread and crackers
	Cakes, muffins and pastry	Scones, pikelets, doughnuts, cakes, sweet buns, pancakes, crepes, muffins (cake-style), slices etc
		Cake, pikelet and pancake dry mixes
		Sweet pastries (fresh, ambient, chilled and frozen)
**Cereal and cereal-based products**	Breakfast cereal	Ready to eat breakfast cereals
		Oats and other breakfast cereals that require heating
		Other processed cereals (e.g. bran)
	Cereal and nut-based bars	Plain, chocolate-topped and yoghurt-topped cereal-based bars
	Noodles	Plain dry noodles
		Savory/flavored dry noodle-based dishes
	Pasta	Canned and ambient pasta and sauce (with and without meat) products (excludes frozen ready meals)
		Packaged fresh pasta with sauce
		Savory/flavored dry pasta-based side dishes
		Plain dry pasta
	Rice	Plain rice
		Savory rice-based side dishes
	Other breakfast products	All breakfast products not included in "Breakfast cereal"
	Other cereal products	Flour and other unprocessed cereals (e.g. polenta, cous cous, bread crumbs, yeast)
**Confectionery**	Chocolate and sweets	Chocolate-based confectionery, sugar-based confectionery
	Chewing gum	All sugar-sweetened and sugar-free chewing gums and bubble gum products
**Convenience foods**	Pizza	Frozen and refrigerated pre-packed pizzas

	Soup	Canned, chilled and ambient soup products
	Ready meals	Frozen, chilled and ambient pre-prepared meals
	Meal kits	Kits with ingredients to put meals together
	Pre-prepared salads and sandwiches	Chilled pre-prepared salads and sandwiches
	Other convenience foods	Other pre-prepared foods not included in above categories
**Dairy**	Cheese	Feta, haloumi, parmesan and other high-salt cheeses
		All types of full and reduced fat cheddar/Colby etc cheese including shredded, block or sliced
		Soft cheeses such as cream cheese, ricotta and cottage cheese
		Processed cheese slices and products
	Cream	Thickened, sour and regular cream products
	Dairy desserts	Dairy-based desserts (e.g. custards, rice puddings)
		Dairy-based dessert mixes (e.g. powders)
	Ice cream and edible ices	Dairy and non dairy-based ice cream varieties and edible ices
	Milk products	Flavored and unflavored dairy milk products
		Flavored and unflavored soymilks
		Flavored and unflavored oat, almond and other milks
		Condensed, evaporated and powdered milk products (including coconut milk)
	Yoghurt products	Fruit, flavored and natural yoghurts (full fat, reduced fat and skim varieties) including yoghurt drinks
**Edible oils**		Salted and unsalted butter and margarine products
**Fruit, vegetables, nuts and legumes**	Fruit	Dried fruit products including coconut
		Fruit-based bars
		Fruit products canned in juice or syrup
		Fruit gels, fruits in jelly and fruit puree
	Jam and fruit spreads	Jams, marmalades and other preserves
	Nuts and seeds	Salted and unsalted nuts and seeds
	Vegetables	Canned tomato products
		Canned beans and peas
		Baked beans in tomato sauce (with and without additions)
		Canned creamed, plain and sweet corn
		All other canned vegetables
		Pickled vegetable and olive products
		Frozen potato-based products
		Frozen vegetables
**Meat and meat alternatives**	Meat alternatives	Plain tofu and other meat-free alternatives
		Meat-free products (e.g. meat-free sausages)
	Processed meat	Pre-packed bacon products
		Beef, pork, chicken and lamb sausages and chilled hot dogs
		Pre-packaged sliced deli meats

		Pre-packaged salami and cured meats<br>Beef, pork, chicken and lamb meat burgers<br>Canned meat products (excluding soup and pasta)<br>Frozen meat pies, sausage rolls and other meat-based pastry products
**Sauces, dressings and spreads**	Mayonnaise and salad dressings	Full and low-fat mayonnaise<br>Oil-based, vinegar-based and other types of salad dressing
	Sauces	Table sauces such as tomato sauces and ketchups, sweet chilli, BBQ sauces<br>Steak, HP and Worcestershire sauces<br>Soy, fish, oyster and other Asian high-salt sauces<br>Mustard products<br>Marinade products<br>Meat accompaniments (e.g. apple, cranberry and mint sauces)<br>Plain and flavored tomato paste products<br>Asian and Indian flavored powdered, ambient and liquid meal-based sauces<br>Ambient and fresh pasta sauces<br>Recipe bases<br>Liquid and powdered gravies and stock
	Spreads	Crunchy and smooth salted and unsalted peanut butter<br>Relishes, chutneys and pickles<br>Other savory spreads (e.g. vegetable spreads)<br>Pâté spreads<br>Sweet spreads<br>Yeast-extract spreads (e.g. vegemite)<br>Chilled and ambient dips and salsa
**Seafood and seafood products**	Canned seafood	All varieties of plain and flavored canned seafood
	Chilled and frozen seafood	Chilled processed fish products (e.g. smoked salmon)<br>Coated frozen fish products (e.g. fish fingers) and uncoated fish products
**Snack foods**		Plain and flavored potato crisps<br>Plain and flavored snack foods<br>Extruded snacks (e.g. cheesy snacks)<br>Plain and flavored corn chips<br>Pretzels, popcorn and other snackfoods<br>Other fried snackfoods (e.g. plantain chips)<br>All varieties of cracker-based snack packs

<u>Supplementary table 2:</u> Traffic light label aggregate scores for US products

		Traffic light label aggregate score		
**Food category**	**n**	**% healthy**	**% unhealthy**	**Mean score**
Beverages	7,075	81.1	18.9	6.3
Bread and bakery products	26,804	9.7	90.3	9.8
Cereal and cereal products	8,855	19.1	80.9	8.6
Confectionery	15,394	27.6	72.4	8.9
Convenience foods	14,375	35.2	64.8	7.7
Dairy	25,815	25.4	74.6	8.5
Edible oils and emulsions	357	0.3	99.7	9.7
Seafood and seafood products	4,876	54.3	45.7	6.8
Foods for specific dietary use	2,053	11.1	88.9	9.8
Fruit, vegetables, nuts and legumes	26,861	55.9	44.1	7.1
Meat and meat products	12,374	23.4	76.6	8.3
Sauces, dressings and spreads	18,805	28.7	71.3	8.2
Snack foods	11,464	5.3	94.7	9.6
TOTAL FOOD	***163,222***	***27.5***	***72.5***	***8.5***
TOTAL BEVERAGE	***11,886***	***66.1***	***33.9***	***6.6***
**TOTAL**	**175108**	**30.1**	**69.9**	**8.4**

<u>Supplementary Table 3:</u> Proportion of US food and beverage products meeting each color coded criteria by food subcategory

		Total Fat			Saturated Fat			Total Sugars			Sodium		
**Food category**	**Total**	**AMBER**	**GREEN**	**RED**	**AMBER**	**GREEN**	**RED**	**AMBER**	**GREEN**	**RED**	**AMBER**	**GREEN**	**RED**
	**n**	**%**	**%**	**%**	**%**	**%**	**%**	**%**	**%**	**%**	**%**	**%**	**%**
**Beverages**	*7075*	*7*	*86*	*7*	*3*	*87*	*10*	*14*	*13*	*74*	*3*	*85*	*13*
Juices	3,612	2	98	0	1	98	0	14	6	80	0	94	5
Coffee and tea	827	4	93	3	5	89	5	13	23	64	1	97	2
Electrolyte drinks	41	0	100	0	0	100	0	41	15	44	0	88	12
Powdered drinks	1,248	27	36	38	6	46	48	4	12	84	12	36	52
Soft drinks	299	0	100	0	0	100	0	5	8	87	1	99	0
Waters	584	2	98	1	1	98	1	28	35	37	0	98	1
Other beverages	464	8	88	4	3	91	6	24	21	55	3	93	4
**Bread and bakery products**	*26,804*	*18*	*13*	*69*	*6*	*31*	*63*	*15*	*25*	*60*	*27*	*10*	*64*
Biscuits and crackers	11,961	8	5	88	6	19	75	8	19	73	29	13	58
Bread	6,719	45	38	17	7	76	17	34	57	9	8	6	87
Cakes, muffins and pastry	8,124	11	5	84	5	13	82	8	9	83	39	10	52
**Cereal and cereal products**	*8,855*	*37*	*22*	*41*	*12*	*53*	*35*	*11*	*31*	*58*	*23*	*23*	*54*
Breakfast cereal	3,938	52	19	29	12	71	17	8	16	76	19	31	51
Cereal and nut-based bars	2,091	16	2	82	13	19	68	3	3	95	47	21	31
Noodles	672	36	42	22	16	63	21	15	79	6	9	43	47
Pasta	1,048	41	31	28	12	40	48	41	55	4	8	2	90
Rice	581	22	75	3	4	92	4	7	91	2	7	10	83
Other breakfast products	479	28	7	65	11	20	68	12	71	17	15	3	81
Other cereal products	46	11	85	4	4	89	7	2	98	0	28	39	33
**Confectionery**	*15,394*	*6*	*32*	*62*	*1*	*34*	*65*	*1*	*2*	*97*	*21*	*72*	*7*
Chewing gum	134	5	90	4	1	91	7	1	15	84	2	97	1
Chocolate and sweets	15,260	6	31	63	1	33	65	1	2	97	21	72	8
**Convenience foods**	*14,375*	*32*	*35*	*33*	*13*	*50*	*37*	*11*	*86*	*4*	*26*	*5*	*69*
Meal kits	162	19	64	18	8	78	14	4	61	35	2	2	96
Pizza	1,810	28	0	72	9	2	89	10	90	0	3	0	97

Food category	Total	Total Fat			Saturated Fat			Total Sugars			Sodium		
		AMBER	GREEN	RED	AMBER	GREEN	RED	AMBER	GREEN	RED	AMBER	GREEN	RED
	n	%	%	%	%	%	%	%	%	%	%	%	%
Pre-prepared salads and sandwiches	1,915	35	10	55	22	35	43	21	74	4	18	7	75
Ready meals	6,483	42	29	29	17	48	36	9	86	4	30	5	65
Soup	3,639	19	73	7	5	84	11	8	89	3	36	6	58
Other convenience foods	366	19	40	41	8	57	36	11	81	7	15	8	77
**Dairy**	*25,815*	*16*	*27*	*57*	*6*	*28*	*66*	*20*	*41*	*39*	*9*	*54*	*37*
Cheese	10,129	4	5	91	1	6	94	9	88	3	8	2	90
Cream	1,511	11	10	79	0	31	69	27	36	37	15	80	5
Desserts	566	25	69	6	31	57	12	5	8	87	53	33	14
Ice cream and edible ices	6,473	20	19	62	5	18	76	4	2	95	15	85	1
Milk products	3,448	46	47	7	16	53	32	54	13	33	3	94	3
Yoghurt products	3,688	16	83	1	12	79	9	43	14	42	1	99	1
**Edible oils**	*357*	*0*	*0*	*100*	*0*	*0*	*99*	*4*	*90*	*6*	*5*	*22*	*73*
**Fruit, vegetables, nuts and legumes**	*26,840*	*4*	*58*	*37*	*2*	*63*	*34*	*13*	*56*	*31*	*22*	*48*	*30*
Fruit	5,528	4	85	10	1	89	10	20	4	75	7	89	4
Jam and fruit spreads	945	3	95	3	1	97	3	2	4	94	5	90	5
Nuts and seeds	8,517	1	1	98	3	3	95	15	53	32	20	41	40
Vegetables	11,850	7	84	9	3	93	5	10	86	4	32	31	37
**Meat and meat products**	*12,374*	*23*	*23*	*54*	*11*	*35*	*54*	*7*	*87*	*7*	*4*	*16*	*80*
Meat alternatives	400	48	23	29	12	79	10	9	88	3	11	32	57
Processed meat	11,974	22	23	55	11	34	55	7	87	7	4	15	81
**Sauces, dressings and spreads**	*18,805*	*11*	*46*	*42*	*6*	*60*	*34*	*22*	*49*	*29*	*11*	*12*	*77*
Mayonnaise and salad dressings	4,182	5	19	75	9	31	60	23	43	33	7	12	82
Sauces	8,682	16	68	16	4	83	13	21	44	35	9	13	78
Spreads	5,941	9	33	58	7	47	45	24	58	17	16	11	73
**Seafood and seafood products**	*4,876*	*25*	*47*	*28*	*17*	*66*	*18*	*3*	*96*	*1*	*25*	*19*	*56*
Canned seafood	1,532	29	41	31	21	56	23	4	95	1	20	5	76
Chilled and frozen seafood	3,344	23	50	27	15	70	15	3	96	1	28	25	47
**Snack foods**	*11464*	*8*	*6*	*86*	*13*	*20*	*67*	*15*	*62*	*23*	*10*	*7*	*83*

Supplementary Table 4: **Nutrient levels per 100g for US products that receive a "Red" traffic light rating**

	All products receiving "Red" traffic light			Products with "Red" traffic lights due to exceeding serving size cut-offs				Products with "Red" traffic lights due to exceeding only per 100g criteria			
	**N**	**Mean**	**Range**	**N**	**%**	**Mean**	**Range**	**N**	**%**	**Mean**	**Range**
Food											
Total fat (g/100g)	87,889	25.1	3.1-100	29,567	34	12.8	3.1-17.5	58,322	66	31.4	17.6-100
Saturated fat (g/100g)	82,943	9.5	0.4-71.4	23,897	29	3.7	0.4-5.0	59,046	71	11.9	5.1-71.4
Sugar (g/100g)	63,710	34.9	1.3-100	18,589	29	16.9	1.3-22.5	45,121	71	42.2	22.6-100
Sodium (mg/100g)	88,093	740	30-31636	47,536	54	429	30-600	40,557	46	1105	601-31636
Beverages											
Total fat (g/100g)	1,540	15.7	2.3-60	92	6	4.6	2.3-8.2	1,448	94	16.4	8.8-60
Saturated fat (g/100g)	2,331	7.6	0.3-50	644	28	2.0	0.3-2.5	1,687	72	9.7	2.6-50
Sugar (g/100g)	7,031	21.7	0.5-100	3,472	49	8.5	0.5-11.25	3,559	51	34.6	11.3-100
Sodium (mg/100g)	1,058	640	78-3000	223	21	211	78-299	835	79	754	302-3000

Supplementary Table 5: Nutrient levels per 100g for US products that receive a "Red" traffic light rating by food subcategory

	All products receiving "Red" traffic light			Products with "Red" traffic lights due to exceeding only per 100g criteria				Products with "Red" traffic lights due to exceeding serving size cut-offs			
	**N**	**Mean**	**Range**	**N**	**%**	**Mean**	**Range**	**N**	**%**	**Mean**	**Range**
Beverages											
Total fat (g/100g)	525	17.4	2.7-56.3	514	98	17.7	8.8-56.3	11	2	4.3	2.7-7.1
Saturated fat (g/100g)	691	11.5	0.3-37.5	649	94	12.1	2.6-37.5	42	6	1.5	0.3-2.5
Sugar (g/100g)	5,204	21.2	0.8-100	2,450	47	35.3	11.3-100	2,754	53	8.6	0.8-11.25
Sodium (mg/100g)	885	684	78-3000	709	80	796	302-3000	176	20	233	78-299
Bread and bakery products											
Total fat (g/100g)	18,459	19.0	3.1-75.0	10,330	56	23.5	17.6-75.0	8,129	44	13.4	3.1-17.5
Saturated fat (g/100g)	16,800	8.4	0.5-52.2	11,981	71	10.2	5.1-52.2	4,819	29	3.8	0.5-5.0
Sugar (g/100g)	16,065	31.8	2.7-97.0	12,954	81	35.2	22.6-97.0	3,111	19	17.6	2.7-22.5
Sodium (mg/100g)	17,029	521	129-3069	3,970	23	839	601-3069	13,059	77	425	129-600
Cereal and cereal products											
Total fat (g/100g)	3,637	17.9	5.5-53.6	1,525	42	25.3	17.6-53.6	2,112	58	12.5	5.5-17.5
Saturated fat (g/100g)	3,084	6.1	1.9-40.7	1,475	48	8.7	5.1-40.7	1,609	52	3.7	1.9-5.0
Sugar (g/100g)	5,138	27.4	8.2-75.0	3,356	65	32.4	22.6-75.0	1,782	35	18.0	8.2-22.5
Sodium (mg/100g)	4,800	682	218-6000	1,940	40	1041	601-6000	2,860	60	439	218-600
Confectionery											
Total fat (g/100g)	9,548	28.9	8.8-100	8,309	87	31.2	17.6-100	1,239	13	13.5	8.8-17.5
Saturated fat (g/100g)	9,987	16.1	0.6-64.3	9,223	92	17.1	5.1-64.3	764	8	3.9	0.6-5.0
Sugar (g/100g)	14,920	53.4	2.6-100	14,566	98	54.3	22.6-100	354	2	17.5	2.6-22.5
Sodium (mg/100g)	1,151	503	302-2422	237	21	914	604-2422	914	79	397	302-600
Convenience foods											
Total fat (g/100g)	4,707	13.7	3.5-64.3	811	17	23.1	17.6-64.3	3,896	83	11.8	3.5-17.5
Saturated fat (g/100g)	5,303	5.0	1.0-24.2	1,870	35	7.4	5.1-24.2	3,433	65	3.6	1.0-5.0
Sugar (g/100g)	565	18.4	7.3-63.3	112	20	31.7	22.7-63.3	453	80	15.1	7.3-22.2
Sodium (mg/100g)	9,945	693	110-29000	2,651	27	1453	601-29000	7,294	73	417	110-600
Dairy											
Total fat (g/100g)	14,686	22.3	2.3-85.7	10,232	70	26.6	8.9-85.7	4,454	30	12.5	2.3-17.5

	All products receiving "Red" traffic light			Products with “Red” traffic lights due to exceeding only per 100g criteria				Products with “Red” traffic lights due to exceeding serving size cut-offs			
	**N**	**Mean**	**Range**	**N**	**%**	**Mean**	**Range**	**N**	**%**	**Mean**	**Range**
Saturated fat (g/100g)	16,952	12.2	0.5-50	14,661	86	13.6	2.6-50.0	2,291	14	3.1	0.5-5.0
Sugar (g/100g)	10,181	21.3	0.5-100	3,986	39	28.9	11.3-100	6,195	61	16.5	0.5-22.5
Sodium (mg/100g)	9,452	784	81-3929	7,066	75	898	325-3929	2,386	25	446	81-600
Edible oils											
Total fat (g/100g)	356	68.9	20-100	356	100	68.9	20.0-100	-	-	-	-
Saturated fat (g/100g)	355	31.2	3.6-71.4	354	100	31.2	7.1-71.4	1	0	3.6	-
Sugar (g/100g)	22	27.4	12.5-47.5	14	64	32.2	24.1-47.5	8	36	17.3	12.5-21.4
Sodium (mg/100g)	259	693	306-1950	209	81	746	607-1950	50	19	471	306-579
Seafood and seafood products											
Total fat (g/100g)	1,378	14.3	7.9-84.9	280	20	22.9	17.6-84.9	1,098	80	12.1	7.9-17.5
Saturated fat (g/100g)	859	4.4	2.5-25.9	182	21	7.9	5.1-25.9	677	79	3.5	2.5-5.0
Sugar (g/100g)	38	15.6	11.5-38.3	3	8	30.1	25-38.3	35	92	14.3	11.5-20.0
Sodium (mg/100g)	2,733	712	265-10625	825	30	1365	602-10625	1,908	70	429	265-600
Fruit, vegetables, nuts and legumes											
Total fat (g/100g)	10,034	40.5	6.5-100	8,779	87	44.4	17.6-100	1,255	13	13.1	6.5-17.5
Saturated fat (g/100g)	9,231	7.1	1.3-66.7	6,110	66	8.7	5.1-66.7	3,121	34	3.8	1.3-5.0
Sugar (g/100g)	8,258	36.8	8.5-95.2	5,612	68	46.8	22.6-95.2	2,646	32	15.6	8.5-22.5
Sodium (mg/100g)	8,042	679	260-31636	2,619	33	1255	601-31636	5,423	67	401	260-600
Meat and meat products											
Total fat (g/100g)	6,731	22.1	5.1-100	4,188	62	27.9	17.6-100	2,543	38	12.6	5.1-17.5
Saturated fat (g/100g)	6,641	8.0	0.4-38.5	4,658	70	9.9	5.1-38.5	1,983	30	3.6	0.4-5.0
Sugar (g/100g)	825	19.8	7.5-57.1	201	24	28.9	22.6-57.1	624	76	16.9	7.5-22.2
Sodium (mg/100g)	9,892	959	30-8375	7,604	77	1108	601-8375	2,288	23	463	30-600
Sauces, dressings and spreads											
Total fat (g/100g)	7,982	32.1	6.8-100	5,802	73	39.3	17.6-100	2,180	27	13.0	6.8-17.5
Saturated fat (g/100g)	6,346	7.1	2.1-64.2	3,841	61	9.3	5.1-64.3	2,505	39	3.8	2.1-5.0
Sugar (g/100g)	5,474	25.6	7.6-100	2,647	48	35.4	22.6-100	2,827	52	16.3	7.6-22.5
Sodium (mg/100g)	14,510	939	216-19286	7,779	54	1366	601-19286	6,731	46	445	216-600

	All products receiving "Red" traffic light			Products with “Red” traffic lights due to exceeding only per 100g criteria				Products with “Red” traffic lights due to exceeding serving size cut-offs			
	**N**	**Mean**	**Range**	**N**	**%**	**Mean**	**Range**	**N**	**%**	**Mean**	**Range**
Snack foods											
Total fat (g/100g)	9,888	26.0	7.6-91.7	8,119	82	28.8	17.6-91.7	1,769	18	13.3	7.6-17.5
Saturated fat (g/100g)	7,640	7.8	1.8-60	4,785	63	10.2	5.1-60	2,855	37	3.7	1.8-5.0
Sugar (g/100g)	2,592	35.1	8.9-98.9	1,948	75	41.3	22.6-98.9	644	25	16.4	8.9-22.5
Sodium (mg/100g)	9,564	751	202-7800	5,733	60	952	601-7800	3,831	40	449	202-600

Supplementary table 6: **Proportion of traffic light color combinations for each major food category**

Food category				Beverages n=7075	Bread and bakery n=26804	Cereal and cereal products n=8855	Confectionery n=15394	Convenience foods n=14375	Dairy n=25815	Edible oils n=357
TOTAL FAT	SAT FAT	SUGAR	SALT	%	%	%	%	%	%	%
AMBER	AMBER	AMBER	AMBER	0	0	0	0	0	0	0
AMBER	AMBER	AMBER	GREEN	0	0	0	0	0	2	0
AMBER	AMBER	AMBER	RED	0	0	1	0	1	0	0
AMBER	AMBER	GREEN	AMBER	0	0	0	0	2	0	0
AMBER	AMBER	GREEN	GREEN	0	0	1	0	0	0	0
AMBER	AMBER	GREEN	RED	0	1	1	0	6	0	0
AMBER	AMBER	RED	AMBER	0	0	1	0	0	0	0
AMBER	AMBER	RED	GREEN	1	0	0	0	0	2	0
AMBER	AMBER	RED	RED	0	0	1	0	0	0	0
AMBER	GREEN	AMBER	AMBER	0	0	0	0	1	0	0
AMBER	GREEN	AMBER	GREEN	0	0	0	0	0	0	0
AMBER	GREEN	AMBER	RED	0	5	1	0	1	0	0
AMBER	GREEN	GREEN	AMBER	0	0	1	0	3	0	0
AMBER	GREEN	GREEN	GREEN	0	0	4	0	0	0	0
AMBER	GREEN	GREEN	RED	0	5	3	0	7	0	0
AMBER	GREEN	RED	AMBER	0	0	4	0	0	0	0
AMBER	GREEN	RED	GREEN	1	0	1	1	0	1	0
AMBER	GREEN	RED	RED	1	1	12[a]	0	0	0	0
AMBER	RED	AMBER	AMBER	0	0	0	0	0	0	0
AMBER	RED	AMBER	GREEN	0	0	0	0	0	3	0
AMBER	RED	AMBER	RED	0	0	1	0	1	0	0
AMBER	RED	GREEN	AMBER	0	0	0	0	2	0	0
AMBER	RED	GREEN	GREEN	0	0	0	0	0	1	0
AMBER	RED	GREEN	RED	0	1	2	0	7	1	0

Food category				Beverages n=7075	Bread and bakery n=26804	Cereal and cereal products n=8855	Confectionery n=15394	Convenience foods n=14375	Dairy n=25815	Edible oils n=357
TOTAL FAT	SAT FAT	SUGAR	SALT	%	%	%	%	%	%	%
AMBER	RED	RED	AMBER	0	1	0	1	0	1	0
AMBER	RED	RED	GREEN	1	0	0	3	0	5	0
AMBER	RED	RED	RED	2	1	2	0	0	0	0
GREEN	AMBER	AMBER	AMBER	0	0	0	0	0	0	0
GREEN	AMBER	AMBER	GREEN	0	0	0	0	0	0	0
GREEN	AMBER	AMBER	RED	0	0	0	0	0	0	0
GREEN	AMBER	GREEN	AMBER	0	0	0	0	0	0	0
GREEN	AMBER	GREEN	GREEN	0	0	0	0	0	0	0
GREEN	AMBER	GREEN	RED	0	0	0	0	0	0	0
GREEN	AMBER	RED	AMBER	0	0	0	0	0	0	0
GREEN	AMBER	RED	GREEN	1	0	0	0	0	1	0
GREEN	AMBER	RED	RED	0	0	0	0	0	0	0
GREEN	GREEN	AMBER	AMBER	0	0	0	0	1	0	0
GREEN	GREEN	AMBER	GREEN	11	0	0	0	0	9	0
GREEN	GREEN	AMBER	RED	2	3	4	0	2	0	0
GREEN	GREEN	GREEN	AMBER	0	1	1	0	13	0	0
GREEN	GREEN	GREEN	GREEN	10	1	3	1	3	3	0
GREEN	GREEN	GREEN	RED	1	5	6	0	14	1	0
GREEN	GREEN	RED	AMBER	1	0	1	3	0	1	0
GREEN	GREEN	RED	GREEN	58[a]	1	1	26	0	10	0
GREEN	GREEN	RED	RED	1	1	5	1	1	0	0
GREEN	RED	AMBER	GREEN	0	0	0	0	0	0	0
GREEN	RED	AMBER	RED	0	0	0	0	0	0	0
GREEN	RED	GREEN	AMBER	0	0	0	0	0	0	0
GREEN	RED	GREEN	GREEN	0	0	0	0	0	0	0
GREEN	RED	GREEN	RED	0	0	0	0	0	0	0

TOTAL FAT	SAT FAT	SUGAR	SALT	Beverages n=7075 %	Bread and bakery n=26804 %	Cereal and cereal products n=8855 %	Confectionery n=15394 %	Convenience foods n=14375 %	Dairy n=25815 %	Edible oils n=357 %
GREEN	RED	RED	AMBER	0	0	0	0	0	0	0
GREEN	RED	RED	GREEN	0	0	0	0	0	0	0
GREEN	RED	RED	RED	0	0	0	0	0	0	0
RED	AMBER	AMBER	AMBER	0	0	0	0	0	0	0
RED	AMBER	AMBER	GREEN	0	0	0	0	0	0	0
RED	AMBER	AMBER	RED	0	1	0	0	0	0	0
RED	AMBER	GREEN	AMBER	0	0	0	0	0	0	0
RED	AMBER	GREEN	GREEN	0	0	0	0	0	0	0
RED	AMBER	GREEN	RED	0	1	0	0	2	0	0
RED	AMBER	RED	AMBER	0	1	2	0	0	0	0
RED	AMBER	RED	GREEN	0	0	2	0	0	0	0
RED	AMBER	RED	RED	0	1	1	0	0	0	0
RED	GREEN	AMBER	AMBER	0	0	0	0	0	0	0
RED	GREEN	AMBER	GREEN	0	0	0	0	0	0	0
RED	GREEN	AMBER	RED	0	1	0	0	0	0	0
RED	GREEN	GREEN	AMBER	0	0	0	0	0	0	0
RED	GREEN	GREEN	GREEN	0	0	0	0	0	0	0
RED	GREEN	GREEN	RED	0	2	0	0	1	0	0
RED	GREEN	RED	AMBER	0	1	2	0	0	0	0
RED	GREEN	RED	GREEN	0	0	1	1	0	0	0
RED	GREEN	RED	RED	0	1	1	0	0	0	0
RED	RED	AMBER	AMBER	0	0	0	0	0	0	0
RED	RED	AMBER	GREEN	0	0	0	0	0	1	1
RED	RED	AMBER	RED	0	3	2	0	2	3	2
RED	RED	GREEN	AMBER	0	1	0	0	2	2	4
RED	RED	GREEN	GREEN	0	0	0	1	0	2	19

TOTAL FAT	SAT FAT	SUGAR	SALT	Beverages n=7075 %	Bread and bakery n=26804 %	Cereal and cereal products n=8855 %	Confectionery n=15394 %	Convenience foods n=14375 %	Dairy n=25815 %	Edible oils n=357 %
RED	RED	GREEN	RED	0	5	6	0	21[a]	29[a]	67[a]
RED	RED	RED	AMBER	1	21	8	15	0	3	1
RED	RED	RED	GREEN	1	5	6	38[a]	0	14	1
RED	RED	RED	RED	5	24[a]	6	5	1	1	3

TOTAL FAT	SAT FAT	SUGAR	SALT	Seafood n=4876 %	Foods for specific dietary use n=2053 %	Fruit and vegetables n=26840 %	Meat and meat alternatives n=12374 %	Sauces and spreads n=18805 %	Snack foods n=11464 %
AMBER	AMBER	AMBER	AMBER	0	0	0	0	0	0
AMBER	AMBER	AMBER	GREEN	0	0	0	0	0	0
AMBER	AMBER	AMBER	RED	0	0	0	1	0	0
AMBER	AMBER	GREEN	AMBER	1	0	0	0	0	0
AMBER	AMBER	GREEN	GREEN	0	0	0	1	0	0
AMBER	AMBER	GREEN	RED	4	0	0	5	1	0
AMBER	AMBER	RED	AMBER	0	2	0	0	0	0
AMBER	AMBER	RED	GREEN	0	1	0	0	0	0
AMBER	AMBER	RED	RED	0	1	0	1	0	0
AMBER	GREEN	AMBER	AMBER	0	0	0	0	0	0
AMBER	GREEN	AMBER	GREEN	0	1	0	0	0	0
AMBER	GREEN	AMBER	RED	1	0	0	1	1	1
AMBER	GREEN	GREEN	AMBER	3	0	0	1	1	0
AMBER	GREEN	GREEN	GREEN	6	2	0	1	0	0

TOTAL FAT	SAT FAT	SUGAR	SALT	Seafood n=4876	Foods for specific dietary use n=2053	Fruit and vegetables n=26840	Meat and meat alternatives n=12374	Sauces and spreads n=18805	Snack foods n=11464
				%	%	%	%	%	%
AMBER	GREEN	GREEN	RED	7	1	1	6	3	3
AMBER	GREEN	RED	AMBER	0	1	0	0	0	0
AMBER	GREEN	RED	GREEN	0	3	0	0	0	0
AMBER	GREEN	RED	RED	0	1	0	2	2	1
AMBER	RED	AMBER	AMBER	0	0	0	0	0	0
AMBER	RED	AMBER	GREEN	0	0	0	0	0	0
AMBER	RED	AMBER	RED	0	0	0	0	0	0
AMBER	RED	GREEN	AMBER	1	0	0	0	0	0
AMBER	RED	GREEN	GREEN	0	0	0	1	0	0
AMBER	RED	GREEN	RED	1	0	0	1	1	0
AMBER	RED	RED	AMBER	0	2	0	0	0	0
AMBER	RED	RED	GREEN	0	0	0	0	0	0
AMBER	RED	RED	RED	0	1	0	0	0	0
GREEN	AMBER	AMBER	AMBER	0	0	0	0	0	0
GREEN	AMBER	AMBER	GREEN	0	0	0	0	0	0
GREEN	AMBER	AMBER	RED	0	0	0	0	0	0
GREEN	AMBER	GREEN	AMBER	0	0	0	0	0	0
GREEN	AMBER	GREEN	GREEN	0	0	0	0	0	0
GREEN	AMBER	GREEN	RED	0	0	0	0	0	0
GREEN	AMBER	RED	AMBER	0	0	0	0	0	0
GREEN	AMBER	RED	GREEN	0	0	0	0	0	0
GREEN	AMBER	RED	RED	0	0	0	0	0	0
GREEN	GREEN	AMBER	AMBER	0	0	1	0	1	0
GREEN	GREEN	AMBER	GREEN	0	1	5	0	2	0
GREEN	GREEN	AMBER	RED	1	0	1	1	6	0

TOTAL FAT	SAT FAT	SUGAR	SALT	Seafood n=4876	Foods for specific dietary use n=2053	Fruit and vegetables n=26840	Meat and meat alternatives n=12374	Sauces and spreads n=18805	Snack foods n=11464
				%	%	%	%	%	%
GREEN	GREEN	GREEN	AMBER	14	0	12	1	3	0
GREEN	GREEN	GREEN	GREEN	11	4	12	9	2	1
GREEN	GREEN	GREEN	RED	20[a]	1	9	10	15[a]	2
GREEN	GREEN	RED	AMBER	0	1	1	0	1	0
GREEN	GREEN	RED	GREEN	0	3	15[a]	0	5	1
GREEN	GREEN	RED	RED	0	1	1	1	12	0
GREEN	RED	AMBER	GREEN	0	0	0	0	0	0
GREEN	RED	AMBER	RED	0	0	0	0	0	0
GREEN	RED	GREEN	AMBER	0	0	0	0	0	0
GREEN	RED	GREEN	GREEN	0	0	0	0	0	0
GREEN	RED	GREEN	RED	0	0	0	0	0	0
GREEN	RED	RED	AMBER	0	0	0	0	0	0
GREEN	RED	RED	GREEN	0	0	0	0	0	0
GREEN	RED	RED	RED	0	0	0	0	0	0
RED	AMBER	AMBER	AMBER	0	0	0	0	0	0
RED	AMBER	AMBER	GREEN	0	0	0	0	0	0
RED	AMBER	AMBER	RED	0	0	0	0	1	2
RED	AMBER	GREEN	AMBER	2	0	0	0	0	1
RED	AMBER	GREEN	GREEN	0	1	0	0	0	0
RED	AMBER	GREEN	RED	7	1	0	2	3	8
RED	AMBER	RED	AMBER	0	2	0	0	0	0
RED	AMBER	RED	GREEN	0	2	0	0	0	0
RED	AMBER	RED	RED	0	1	0	0	1	1
RED	GREEN	AMBER	AMBER	0	0	0	0	0	0
RED	GREEN	AMBER	GREEN	0	0	0	0	0	0

TOTAL FAT	SAT FAT	SUGAR	SALT	Seafood n=4876 %	Foods for specific dietary use n=2053 %	Fruit and vegetables n=26840 %	Meat and meat alternatives n=12374 %	Sauces and spreads n=18805 %	Snack foods n=11464 %
RED	GREEN	AMBER	RED	0	0	0	0	1	2
RED	GREEN	GREEN	AMBER	0	0	0	0	0	0
RED	GREEN	GREEN	GREEN	0	0	0	0	0	0
RED	GREEN	GREEN	RED	2	0	2	1	3	5
RED	GREEN	RED	AMBER	0	1	0	0	0	0
RED	GREEN	RED	GREEN	0	1	0	0	0	0
RED	GREEN	RED	RED	0	1	0	0	1	1
RED	RED	AMBER	AMBER	0	3	1	0	1	0
RED	RED	AMBER	GREEN	0	0	1	0	1	0
RED	RED	AMBER	RED	1	4	3	3	8	9
RED	RED	GREEN	AMBER	4	2	2	1	1	2
RED	RED	GREEN	GREEN	1	1	7	4	1	1
RED	RED	GREEN	RED	9	5	9	41[a]	14	37[a]
RED	RED	RED	AMBER	0	15	4	0	1	3
RED	RED	RED	GREEN	0	7	5	0	1	2
RED	RED	RED	RED	0	26[a]	2	1	5	11

[a] Most common combination
